# Mayonnaise Enriched with Flaxseed Oil: Omega-3 Fatty Acids Content, Sensory Quality and Stability during the Storage

**DOI:** 10.3390/foods11152288

**Published:** 2022-07-31

**Authors:** Mohammed El-Waseif, Badr Saed, Hany Fahmy, Ahmed Sabry, Hamdy Shaaban, Mohamed Abdelgawad, Ali Amin, Amr Farouk

**Affiliations:** 1 Food Science and Technology Department, Faculty of Agricultural, Al-Azhar University, Cairo 11651, Egypt; elwaseif@azhar.edu.eg (M.E.-W.); badrsaed39.el@azhar.edu.eg (B.S.); hany.fahmy@azhar.edu.eg (H.F.); ahmedsabry0080@azhar.edu.eg (A.S.); 2 Flavour and Aroma Chemistry Department, National Research Center, Cairo 12622, Egypt; ha.shaaban@nrc.sci.eg; 3 Department of Pharmaceutical Chemistry, College of Pharmacy, Jouf University, Sakaka 72341, Saudi Arabia; 4 Deanship of Scientific Research, Umm Al-Qura University, Makkah 21955, Saudi Arabia; ahamin@uqu.edu.sa; 5 Zoology Department, Faculty of Science, Mansoura University, Mansoura 35516, Egypt

**Keywords:** oxidative stability, microbiological growth, peroxide value, free fatty acids, p-anisidine value

## Abstract

This study aimed to produce healthy mayonnaise with a protective effect against cardiovascular diseases, containing omega-3 fatty acids (FA), using flaxseed oil (FXO), which includes a high percentage of alpha-linolenic acid (ALA, C18:3n-3). The mayonnaise was prepared by replacing soybean oil with FXO at 20, 30, and 40% levels. The effect on the organoleptic, physical, and chemical quality was studied compared to a control, prepared only with soybean oil (70%). The oxidative and microbial stability during 12 weeks of storage at 25 and 7 °C was also evaluated. The results showed that the use of FXO in mayonnaise (20, 30, and 40%) led to an increase in TUFA (from 79.37 (control) to 82.48, 85.49, and 87.66%, respectively), particularly in PUFAn-3, due to the rise of ALA (from 6.5 to 18.38, 24.02 and 37.87%, respectively) and a decrease in TSFA (from 20.63 to 17.52, 14.51 and 12.34%, respectively). The panelists did not perceive significant differences in the sensory characteristics of the “new” mayonnaise. A decrease in the oxidation rates of the “new” mayonnaise during the storage period was observed. A significant effect on microbial growth was not reported, although the permissible limits were not exceeded after 12 weeks of storage, even at 25 °C.

## 1. Introduction

Mayonnaise is one of the most popular sauces globally, with a relatively long shelf life of weeks or months depending on refrigerated storage conditions [1]. Traditional mayonnaise is an oil-in-water emulsion with a 70–80% dispersed lipid phase, prepared by emulsifying edible oil with egg yolk in an aqueous phase with vinegar, salt, and seasonings. The most common oils used in traditional mayonnaise formulation are soybean, sunflower, corn, and rapeseed oils; however, due to economic and industrial issues, most studies used soybean oil as a reference [2].

Due to the high oil content with its high content of unsaturated fatty acids, lipid oxidation is predicted to be initiated at the oil-aqueous interface and increases in the oil phase during storage [3]. The formation of lipid-free radicals due to the oxidation process negatively affects the sensory and nutritional properties of the mayonnaise, especially during storage with discoloration and toxic components generation. Therefore, many studies have shown the enrichment of mayonnaise with natural antioxidants to keep the product healthy and stable [4]. In addition, increased dietary fat intake is associated with increased risks of high cholesterol, obesity, cardiovascular disease, some types of cancer, and gallbladder disease, leading to a trend toward low-fat food products [5]. Accordingly, using healthier oils in mayonnaise—such as virgin coconut oil, which contains medium-chain fatty acids, fish oil rich in polyunsaturated fatty acids, or reducing the fat content by using gums—leads to innovative, stable, and functional food products [6,7,8,9].

Flaxseed (*Linum usitatissimum*) oil has gained popularity in the natural foods market due to its health benefits, including preventing coronary heart disease, certain cancer types, neurological and hormonal disorders, blood pressure reduction, and bad cholesterol (LDL) reduction in humans. Including ω-3 fatty acid or α-linolenic acid (ALA) in flaxseed oil is responsible for most of its health advantages. Flaxseed oil contains the most ALA, accounting for roughly 55–60% of total fatty acids and significant amounts of phenolic acids and tocopherols, representing natural antioxidants [10,11]. It is a tremendous challenge to develop flaxseed oil-based products, such as mayonnaise, due to their higher ALA content. Due to the high content of ALA in flaxseed oil, it has recently attracted greater public interest as a nutritional supplement. There are a variety of flaxseed oil-based goods on the market in the form of nutraceuticals or regular foods, such as oil blends, oil yogurt, solid oil drinks, and so on [11].

Therefore, the current study focuses on incorporating flaxseed oil at different levels (20, 30, and 40%) into mayonnaise to produce a functional product for health benefits. The balance between saturated and unsaturated oil would achieve the health-promoting target. Fatty acids composition and sensory quality were compared with the mayonnaise control, prepared using only soybean oil. Furthermore, the oxidative stability and microbiological growth were evaluated during 12 weeks of ambient temperature and cold storage.

## 2. Materials and Methods

### 2.1. Materials

Soybean oil, eggs, sugar, vinegar, and salt were purchased in Cairo, Egypt’s local market. Boron trifluoride, methanol, sodium hydroxide, sodium thiosulfate, and starch were obtained from Sigma Aldrich Chemical Co. (St. Louis, MO, USA). Plate count agar medium, yeast extract glucose chloramphenicol agar medium, lauryl sulfate tryptose broth, EC broth, half-Fraser broth, tryptone soybean broth, Baird–Parker agar, buffer peptone water, Muller Kuffman tetrathionate novobiocin broth, and xylose-lysine deoxycholate agar were purchased from BD Difco Dehydrated Culture Media (Berkshire, UK).

### 2.2. Flaxseed Oil Extraction

The flaxseeds (*Linum usitatissimum* L.) (Sakha) free from garden cress were obtained from Fiber Crops Research Section, Field Crops Research Institute, Agriculture Research Center, Cairo, Egypt. Flaxseeds were crushed using a laboratory mill, then specimens were compressed by means of a Carver hydraulic press under a pressure of 10,000 lb for 1 h at room temperature according to the method of Ustun et al. [12] and then was filtered, kept in dark brown bottles and stored in a deep freezer at −18 °C until analysis and formulation.

### 2.3. Preparation of Mayonnaise

According to Shinn et al. [13], the control mayonnaise sample was made with 70% soya bean oil, 15% vinegar, 10% egg yolk, 3.0% sugar, and 2% sodium chloride. In a bowl with a third of the total vinegar amount, mayonnaise was prepared until it became a creamy paste. Egg yolk was added and mixed for 5 min. Soybean oil was then slowly added under continuous mixing to form an emulsion, and mixing continued for 5 min. The remaining vinegar was added and mixing continued for an additional 5 min. Samples were stored at ambient (25 ± 5 °C) and cold (7 ± 2 °C) temperatures. Samples were analyzed every two weeks up to 12 weeks according to Azhagu Saravana Babu et al. [14]. The mayonnaise was prepared by replacing soybean oil with flaxseed oil at different levels of 20% (MFXS1), 30% (MFXS2), and 40% (MFXS3).

### 2.4. Lipid Extraction from Mayonnaise

Lipid was extracted from mayonnaise samples according to Park et al. [15]. To break the emulsion, the mayonnaise was frozen for 24 h and then thawed for 2 h at room temperature. The thawed mayonnaise was centrifuged for 10 min at 18,000× *g* at 15 °C (2236R, Gyrozen Co., Daejeon, Korea). The isolated lipid phase was directly employed for subsequent research on lipid oxidative stability in mayonnaise.

### 2.5. Fatty Acids Composition

Gas chromatography was used to determine the methyl esters of the fatty acids in the investigated oils and the lipid isolated from mayonnaise. According to the AOAC [16], the methyl ester samples were produced using boron trifluoride (BF_3_) in methanol (20%) as a methylating agent. Methylated fatty acid analysis was performed on an Agilent 6890 (Agilent, USA) gas chromatograph equipped with a flame ionization detector (GC-FID), using an HP-INNO Wax capillary column (30 m × 0.53 mm × 1 μm, Agilent, Santa Clara, CA, USA). The injection and detector temperatures were set to 280 °C. The gas flow rates used were 30 mL/min for carrier gas (H_2_), 15 mL/min for make-up gas (N_2_), and 300 mL/min for air. The split ratio was 8:1 in duplicate, and 1 μL of the sample solution was injected. The column was ramped at 10 °C/min from 100 to 240 °C and maintained for 10 min. Methylated fatty acids were identified by comparing their retention times to authentic standards mixture C_4_–C_24_ (Supelco, Bellefonte, PA, USA) analyzed under the same conditions. Each sample was analyzed in triplicate and the obtained results were presented as the percentage of the total peak area (% area).

### 2.6. Oxidative Stability of Lipids in Mayonnaise

Progression of lipid oxidation in mayonnaise was monitored by determining free fatty acids content (FFA%), peroxide value (PV), and p-anisidine value (p-AV) [16]. Free fatty acids % (as oleic acid) of the pure vegetable oils, blended oils, and lipid extracted from prepared fat spreads were determined. Before titration against 0.1 (M) NaOH, the tested sample was dissolved in neutralized ethanol-diethyl ether solvent (1:1 *v*/*v*). Lipid hydroperoxide, measured as peroxide value (PV), was determined by the liberated iodine, titrated with 0.01 M sodium thiosulfate solution using a starch solution (1%) as an indicator. The peroxide value was expressed as milliequivalents (meq) of peroxide oxygen per kg of oil. According to the method disclosed, the optical density of the p-anisidine value was measured at 350 nm in a 1 cm cuvette of a solution comprised of 1.00 g of the fat in 100 mL of a mixture of solvent and reagent. The amount of aldehydes (mostly 2–alkenals and 2, 4–dienals) in animal and vegetable fats and oils is determined by reacting the acetic acid solution of aldehydic compounds in the oil with p-anisidine and measuring the absorbance at 350 nm. All samples were analyzed in triplicate.

### 2.7. pH Value

The pH values of the mayonnaise samples were measured using a Testo 230 digital pH meter with a glass electrode (Testo Ltd., Lenzkirch, Germany), according to AOAC [16].

### 2.8. Microbiological Analysis

Total plate bacterial counts: These were determined using plate count agar medium by means of the pour plate technique and then incubated at 30 °C for three days according to the procedures described in ISO 4833-1 [17].

Mold and yeast count: The ISO 6611procedures [18] were followed to determine mold and yeast counts using yeast extract glucose chloramphenicol agar medium. The plates were incubated at 25 °C for 5 days.

*Escherichia coli* detection: The ISO 7251 procedures [19] were followed to detect and enumerate presumptive *Escherichia coli* using lauryl sulfate tryptose broth and EC broth incubated at 44 °C for 48 h.

*Listeria monocytogenes* was detected using half-Fraser broth (selective enrichment medium) and incubated at 30 °C for 24 h, Fraser broth and incubation at 37 °C for 24 h and then plated out on Palcam agar and incubated at 30 °C for 24 h according to the procedures described in ISO 11290-1 [20].

*Staphylococcus aureus* was detected using tryptone soybean broth and Baird–Parker agar and incubated at 37 °C for 48 h, according to the procedures described in USP 43-NF 25 [21].

*Salmonella* was detected using buffer peptone water and Muller Kuffman tetrathionate novobiocin broth, and then xylose-lysine deoxycholate agar incubated at 37 °C for 24 h according to the procedures described in ISO 6579-1 [22].

### 2.9. Sensory Evaluation

Sensory assessment of the manufactured mayonnaise samples was tested following the preparation according to Puligundla [9]. The sensory panel was composed of 4 women and 6 men, ranging in age from 22 to 30, and who were volunteer participants from the technical staff of the Department of Food Science and Technology, Faculty of Agriculture, Azhar University. The samples weighing about 10 g were presented to the panelists at room temperature on coded white plastic saucers. After observing the samples’ structure and appearance (including color), the panelists were instructed to taste the mayonnaise and assess other attributes (flavor, consistency, mouthfeel, spreadability) and overall acceptability using a ten-point hedonic scale, with 1 indicating extreme dislike, 5 indicating neither like nor dislike, and 10 indicating extreme like. The order in which the samples were presented was randomized. All samples were analyzed in triplicate. The order in which the samples were presented was randomized. All samples were analyzed in triplicate.

### 2.10. Statistical Analysis

IBM SPSS Statistics [23] (IBM Software, Somers, NY, USA) was used for statistical analysis. All data are reported in terms of means and standard deviations. The significance threshold was set at *p* < 0.05 using one-way ANOVA followed by Duncan’s test.

## 3. Results and Discussion

### 3.1. Fatty Acids Composition of Mayonnaise Samples

Studies have shown that regular intake of saturated fatty acids increases cholesterol, associated with increased mortality due to coronary heart disease [24,25]. The data in Table 1 showed that the contents of saturated fatty acids were 16.34 and 9.72% for soybean and flaxseed oils, respectively. While they were 20.63, 17.52, 14.51, and 12.34% for the control sample, and mayonnaise supplemented with flaxseed oil at 20%, 30%, and 40%, respectively. Palmitic acid (C16:0) was the most common saturated fatty acid present in all mayonnaise samples, with concentrations ranging from 5 to 13.20%, followed by stearic acid (C18:0) with 4.18–4.45%. Generally, a decrease in total saturated fatty acids content (TSFA) to 17.52, 14.51, and 12.34% for MFXS1, MFXS2, and MFXS3 samples, respectively. The previous findings agree with Nazari et al. [26], who surveyed mayonnaise products in Iran and found TSFA with 18.1–24.9% in the examined products.

According to Satchithanandam et al. [27], trans fatty acids in mayonnaise produced in the USA ranged from 0.0 to 2.2%, which is in line with our findings where no trans fatty acids were detected in the examined samples. The fatty acid ALA (ω3-C_18:3_) showed the highest value among polyunsaturated fatty acids. It represented 6.50% for the control mayonnaise, and this was increased in the mayonnaise samples supplemented with flaxseed oils to 18.38, 24.02, and 37.87% of the total fatty acids in the MFXS1, MFXS2, and MFXS3 samples, respectively. Similar results were obtained using flaxseed oils to formulate therapeutic fat spreads [28]. Table 1 shows that the mayonnaise samples MFXS1, MFXS2, and MFXS3 showed a significant increase in total unsaturated fatty acids (TUFA) content (82.48, 85.49, and 87.66%, respectively). The current study investigated two different indexes: the index of atherogenicity (IA) and the index of thrombogenicity (IT) (Table 1) [29]. The IT index represents the relationship between the sum of the main saturated fatty acids and the sum of the main classes of unsaturated fatty acids. Therefore, consuming foods or products with a lower IA can reduce total cholesterol and LDL-C levels in human blood plasma. In comparison, the IT index measures the propensity of blood arteries to produce clots. The lowering in both IA and IT indexes as well as the increase of the PUFAn-3/PUFAn-6 ration (balance of omega-3 and omega-6 fatty acids) for samples MFXS1, 2, and 3, reflect the importance of using flaxseed oil to prepare healthier mayonnaise. Finally, from the nutrition point of view, replacing soybean oil with 20–40% of flaxseed oil in mayonnaise results in a sauce which is also a better source of α-linolenic acid [30,31].

**Table 1:** Fatty acid profiles of vegetable oils and mayonnaise samples.

Fatty Acid % *	Vegetable Oils **		Mayonnaise Samples ***			
	SBO	FXO	Control Sample	MFXS1	MFXS2	MFXS3
C14:0	0.04	ND	0.80 ± 0.02	0.02 ± 0.01	ND	ND
C16:0	11.11	5.00	13.20 ^a^ ± 0.01	11.51 ^b^ ± 0.03	9.20 ^c^ ± 0.00	7.39 ^d^ ± 0.01
C16:1 (n-9)	0.12	0.01	0.10 ^a^ ± 0.01	0.11 ^a^ ± 0.02	0.07 ^b^ ± 0.02	0.04 ^c^ ± 0.01
C18:0	4.22	4.45	4.34 ^a^ ± 0.04	4.20 ^b^ ± 0.01	4.20 ^b^ ± 0.01	4.18 ^b^ ± 0.03
C18:1 (n-9)	24.20	14.07	22.15 ^b^ ± 0.03	18.25 ^d^ ± 0.05	23.15 ^a^ ± 0.05	20.30 ^c^ ± 0.04
C18:2 (n-6)	52.02	18.47	50.27 ^a^ ± 0.01	45.44 ^b^ ± 0.01	38.25 ^c^ ± 0.02	29.45 ^d^ ± 0.05
C18:3 (n-3)	7.01	57.50	6.50 ^d^ ± 0.02	18.38 ^c^ ± 0.02	24.02 ^b^ ± 0.01	37.87 ^a^ ± 0.02
C20:0	0.61	0.27	1.47 ^a^ ± 0.03	1.15 ^b^ ± 0.05	0.69 ^c^ ± 0.03	0.42 ^d^ ± 0.02
C20:1 (n-9)	0.31	0.23	0.35 ^a^ ± 0.02	0.30 ^b^ ± 0.03	0.30 ^b^ ± 0.01	0.28 ^b^ ± 0.02
C22:0	0.36	ND	0.82 ^a^ ± 0.02	0.64 ^b^ ± 0.04	0.12 ^c^ ± 0.02	0.07 ^c^ ± 0.03
TSFAs ****	16.34	9.72	20.63 ^a^ ± 0.01	17.52 ^b^± 0.02	14.51 ^c^ ± 0.01	12.34 ^d^ ± 0.02
TUFAs	83.66	90.28	79.37 ^d^ ± 0.01	82.48 ^c^ ± 0.02	85.49 ^b^ ± 0.04	87.66 ^a^ ± 0.04
MUFA	24.63	14.31	22.60 ^b^ ± 0.02	18.66 ^d^ ± 0.01	23.52 ^a^ ± 0.03	20.62 ^c^ ± 0.02
PUFA	59.03	75.97	56.77 ^c^ ± 0.03	63.82 ^b^ ± 0.04	62.27 ^b^ ± 0.03	67.32 ^a^ ± 0.01
PUFAn-3/PUFA-n6	0.13	3.11	0.13 ^d^ ± 0.01	0.40 ^c^ ± 0.02	0.62 ^b^ ± 0.03	1.29 ^a^ ± 0.01
AI *****	0.13	0.06	0.20 ^a^ ± 0.03	0.14 ^b^ ± 0.01	0.11 ^c^ ± 0.02	0.08 ^d^ ± 0.01
TI	0.26	0.05	0.32 ^a^ ± 0.02	0.17 ^b^ ± 0.01	0.13 ^c^ ± 0.03	0.08 ^d^ ± 0.02

* The results are expressed as relative percentages after dividing the normalized peak area of each fatty acids by the sum of normalized peak areas for all the fatty acids obtained by GC analysis. Values with different small letters in the same row are significantly different (*p* < 0.05). ** SBO: Soybean oil, FXO: flaxseed oil, *** control sample: 70% SBO, MFXS1: 20% FXO and 50% SBO, MFXS2: 30% FXO and 40% SBO, and MFXS3: 40% FXO and 30% SBO, ND: not detected. **** TSFAs: total saturated fatty acids, TUFAs: total unsaturated fatty acids, MUFA: monounsaturated fatty acids, and PUFA: polyunsaturated fatty acids. ***** Atherogenic index (AI) and thrombogenic index (TI) were calculated according to Ulbricht and Southgate [29].

### 3.2. Sensory Quality of Mayonnaise Samples

From the tabulated results, it can be seen that there were no significant differences (*p* < 0.05) in the values of sensory characteristics assessed for the mayonnaise samples supplemented with flaxseed oil compared to the control sample (Table 2). Thus, the use of flaxseed oil in the formulation process had no significant effect (*p* < 0.05) on the sensory characteristics.

### 3.3. pH Value of Mayonnaise

**Figure 1:**
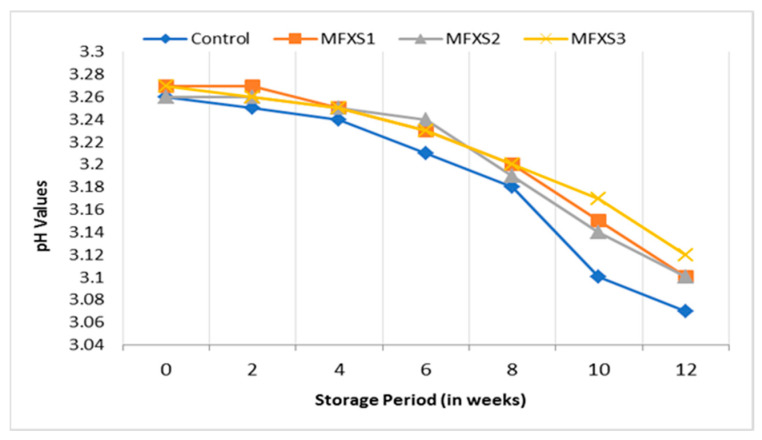
pH values of mayonnaise samples during ambient storage (25 ± 5 °C). Control sample: 70% soy bean oil, MFXS1: 20% flaxseed oil and 50% soy bean oil, MFXS2: 30% flaxseed oil and 40% soy bean oil, and MFXS3: 40% flaxseed oil and 30% soy bean oil.

**Figure 2:**
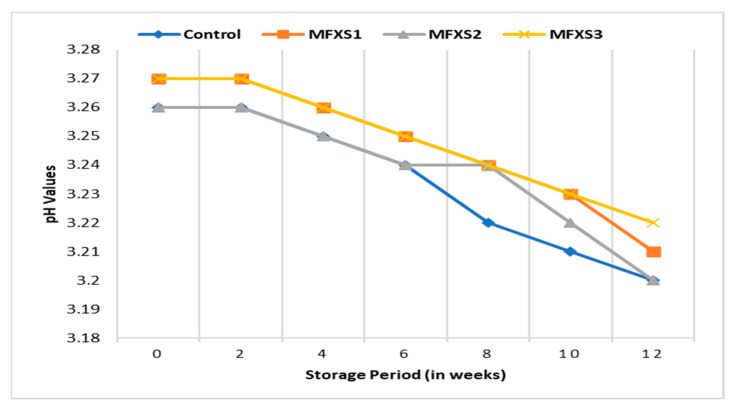
pH values of mayonnaise samples during cold storage (7 ± 2 °C). Control sample: 70% soy bean oil, MFXS1: 20% flaxseed oil and 50% soy bean oil, MFXS2: 30% flaxseed oil and 40% soy bean oil, and MFXS3: 40% flaxseed oil and 30% soy bean oil.

The data presented in Figure 1 and Figure 2 show slight differences in pH values between all studied mayonnaise samples made with different levels of flaxseed oil and soybean oil during storage. With the increase in the storage period, the pH values for all studied samples gradually decreased, and the values ranged from 3.07 to 3.12 at the end of the storage period at the ambient temperature, while they were from 3.20 to 3.22 at the end of the storage period at the cold temperature. This decrease may be due to the growth of lactic acid bacteria, which leads to an increase in acidity and thus a reduction in pH values during the storage period, which is in agreement with Marinescu et al. [32].

Mayonnaise’s structure and stability are heavily influenced by its pH. Acidic pH enhances mayonnaise’s stability by limiting microbial growth, preserving the mayonnaise’s structure, and improving its stability. Mayonnaise has a pH range of 3.6–4.0, with the best viscoelasticity and stability achieved when the pH is close to the egg yolk’s isoelectric point because the proteins have the least charge [33]. The pH affects the distribution of volatile compounds (secondary oxidation products) and acts as a pro-oxidant. Reduced pH from neutral to roughly four can have a significant pro-oxidant effect on mayonnaise by breaking bridges between the egg yolk proteins (low-density lipoproteins, lipovitellin, and phosvitin) and iron. As a result, the egg yolk’s iron is liberated, making it more available as an oxidation initiator. Because weak linkages between proteinous emulsifiers under acidic conditions allow carbonyl molecules (propanal) to easily migrate from the liquid to the gas phase at pH 4, mayonnaise flavor stability may differ significantly due to oxidative stability [34].

### 3.4. Oxidative Stability of Lipids in Mayonnaise Samples

**Figure 3:**
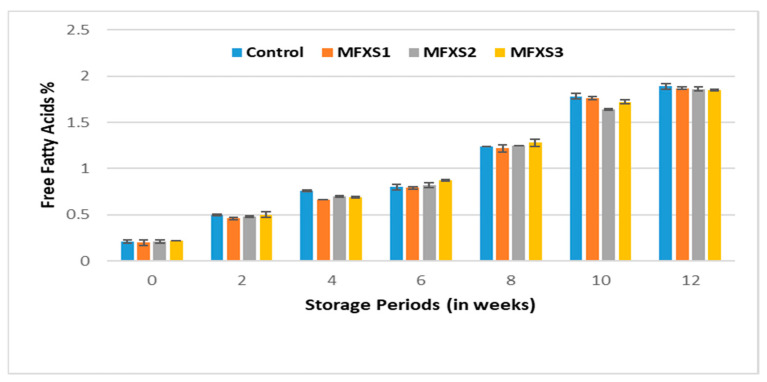
The free fatty acid of mayonnaise samples during ambient storage (25 ± 5 °C). Control sample: 70% soy bean oil, MFXS1: 20% flaxseed oil and 50% soy bean oil, MFXS2: 30% flaxseed oil and 40% soy bean oil, and MFXS3: 40% flaxseed oil and 30% soy bean oil.

**Figure 4:**
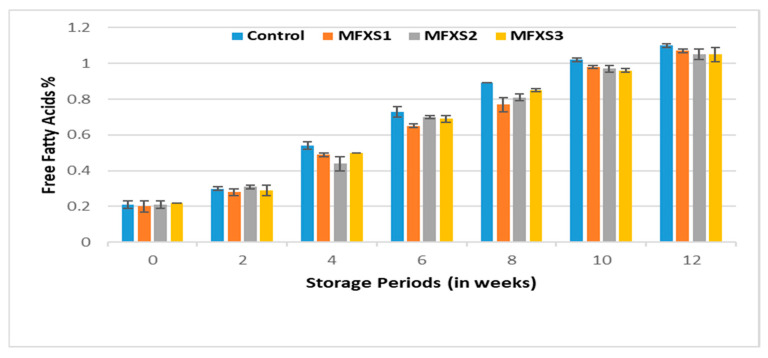
The free fatty acid of mayonnaise samples during cold storage (7 ± 2 °C). Control sample: 70% soy bean oil, MFXS1: 20% flaxseed oil and 50% soy bean oil, MFXS2: 30% flaxseed oil and 40% soy bean oil, and MFXS3: 40% flaxseed oil and 30% soy bean oil.

Oxidative stability of lipids in mayonnaise was determined during storage for 12 weeks under room temperature (25 °C) and cold conditions (7 °C). The FFA% of oils have been used to measure hydrolysis and oxidation, leading to the formation of free fatty acids. The data in Figure 3 and Figure 4 indicate that the FFA% values of the oil extracted from the mayonnaise samples before storage ranged from 0.20 to 0.21%, and the slight increase in these values for the extracted oils can be attributed to the high FFA% value of the egg fat. During storage, the values were as follows: 1.87, 1.86, 1.85, and 1.89% for oils extracted from MFXS1, MFXS2, MFXS3, and control samples, respectively, stored at ambient temperature, while 1.07, 1.05, 1.05 and 1.10% were the FFA% for stored samples at cold temperature, respectively.

The FFA% values of the extracted oils increased gradually and significantly during the storage period, reaching their maximum after 12 weeks, and this could be attributed to the increase in the polyunsaturated fatty acids content of the oils used in the preparation of mayonnaise. The use of flaxseed oil decreased FFA significantly compared to the control sample, especially under cold storage conditions. In addition to the oxidation of the fat phase of mayonnaise, hydrolysis of triglycerides to form free fatty acids is the main reason for increasing FFA% upon storage, as reported by Kaur et al. [35]. The enzyme lipase also breaks down triglycerides as well as mono- and di-lipids. In the advanced oxidative state, FFA% of low molecular weight are developed through the accumulation of acid cleavage products, and thus FFA% increases. This oxidation can also occur with the help of oxidizing enzymes and the presence of a percentage of atmospheric oxygen in the packaging space [36]. The relatively lower FFA content in the products containing antioxidants indicated the marginal resistance to the hydrolysis of the triglycerides [35].

**Figure 5:**
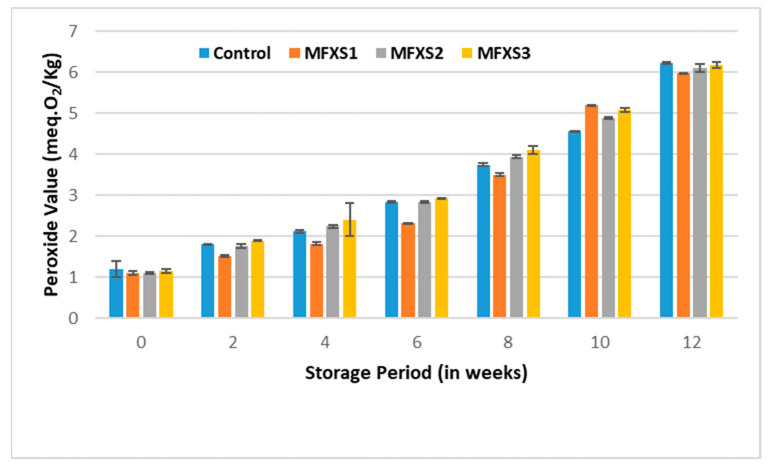
Peroxide value of mayonnaise samples during ambient storage (25 ± 5 °C). Control sample: 70% soy bean oil, MFXS1: 20% flaxseed oil and 50% soy bean oil, MFXS2: 30% flaxseed oil and 40% soy bean oil, and MFXS3: 40% flaxseed oil and 30% soy bean oil.

**Figure 6:**
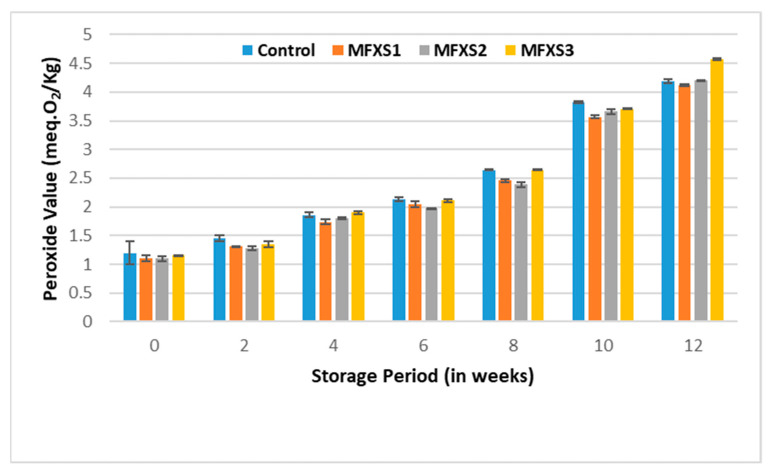
Peroxide values of mayonnaise samples during cold storage (7 ± 2 °C). Control sample: 70% soy bean oil, MFXS1: 20% flaxseed oil and 50% soy bean oil, MFXS2: 30% flaxseed oil and 40% soy bean oil, and MFXS3: 40% flaxseed oil and 30% soy bean oil. Primary oxidizing chemicals, peroxides, and hydroperoxides are unstable intermediates that break up into different carbonyls and other molecules. The p-AV test evaluates the amount of aldehydes in the oil, primarily 2-alkenes, which suggest secondary oxidizing chemicals production [42]. With storage time, the p-AV of lipids in all mayonnaises steadily increased (Figure 7 and Figure 8), related to the formation of secondary oxidation products due to peroxide breakdown [37]. The p-AV, on the other hand, is proportional to unsaturated fatty acid double bonds, with the higher the degree of unsaturation, the larger the oxidation processes [43].

The PV was estimated for the prepared mayonnaise samples and the control sample at the beginning of the storage period and periodically during ambient and cold storage. The data in Figure 5 and Figure 6 show that the PVs of oils extracted from mayonnaise samples increased significantly by increasing the storage period. The increasing rate of PVs for the mayonnaise samples stored at an ambient temperature is higher than the rising rate of PVs for the mayonnaise samples stored at a cold temperature for 12 weeks. It reached its highest levels after 12 weeks. These values were as follows: 5.98, 6.10, 6.17, and 6.22 meq.O_2_/kg for oils extracted from MFXS1, MFXS2, MFXS3, and control sample stored at ambient temperature, while PVs were 4.12, 4.20, 4.57, and 4.19 meq.O_2_/kg when stored at cold temperature, respectively. This could be due to the degradation of peroxides into secondary oxidative products [33,37]. The oxidation of lipids is accelerated by the reactions on the surface of the oil–water emulsion droplets [38]. The previous results and trend of increase in PV are in agreement with the findings of Nour [39] and Park et al. [15].

The PVs for mayonnaise samples fortified with flaxseed oil stored at room temperature were significantly lower than the control, suggesting that the phenolic and flavonoid content in flaxseed oil might retard oxidation in the early stages. It is noteworthy that PVs for samples stored under cold conditions are much lower than those stored under ambient conditions with significant differences. According to Yi et al. [40], flavonoids can increase the oxidative stability of lipids in an O/W emulsion, suggesting that they may contribute to the retardation of lipid oxidation in mayonnaise. The results showed a statistically significant difference (*p* < 0.05) between the initial, at time zero, and the end of the storage period at 12 weeks for all samples stored under ambient or cold conditions. However, all the obtained values were within the permissible limits of the Egyptian standard specifications (less than 15 meq.O_2_/kg oil) [41].

**Figure 7:**
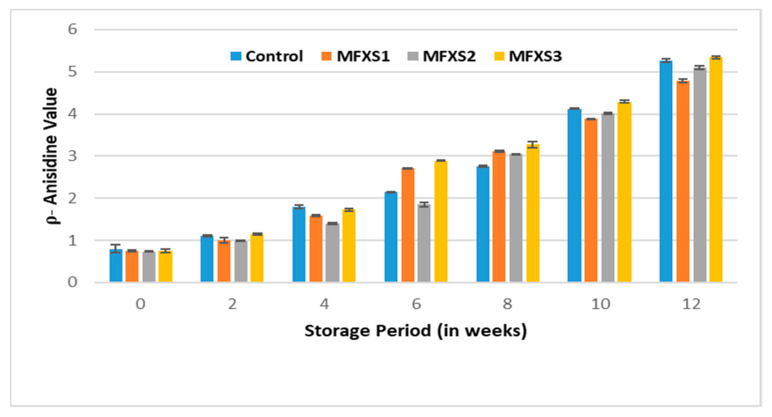
Anisidine value of mayonnaise samples during ambient storage (25 ± 5 °C). Control sample: 70% soy bean oil, MFXS1: 20% flaxseed oil and 50% soy bean oil, MFXS2: 30% flaxseed oil and 40% soy bean oil, and MFXS3: 40% flaxseed oil and 30% soy bean oil.

**Figure 8:**
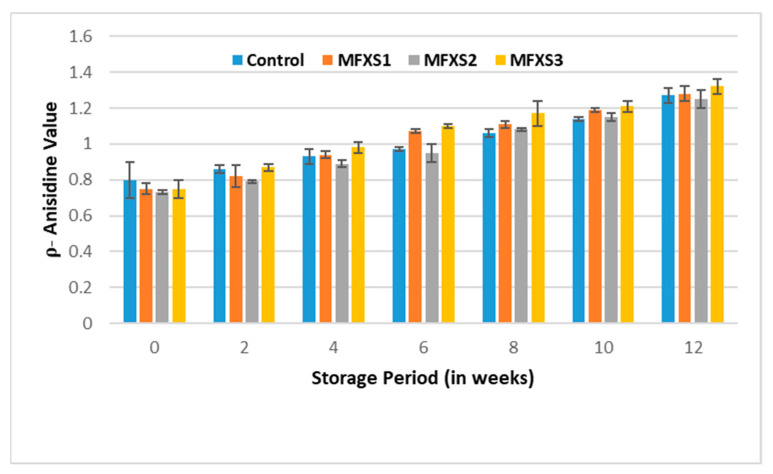
Anisidine value of mayonnaise samples during cold storage (7 ± 2 °C). Control sample: 70% soy bean oil, MFXS1: 20% flaxseed oil and 50% soy bean oil, MFXS2: 30% flaxseed oil and 40% soy bean oil, and MFXS3: 40% flaxseed oil and 30% soy bean oil.

### 3.5. Microbial Stability of Mayonnaise Samples

Significant differences could be observed over the storage periods for all samples, with lower values for samples stored under cold conditions than those stored under ambient ones. From data in Figure 7 and Figure 8, it could be observed that the p-AV for MFXS1, MFXS2, MFXS3, and control samples at the beginning of the storage period were 0.75, 0.73, 0.75, and 0.80, respectively, then significantly (*p* < 0.05), they gradually increased as the storage period increased up to 12 weeks. At the end of the storage period, the highest values of p-AV for the stored samples at ambient temperature ranged from 4.79 to 5.34, while the highest values for the cold-stored samples were between 1.25 and 1.32. Generally, all the obtained values were within the permissible limits of the Egyptian standard specifications (less than 10) [41].

The mayonnaise samples were free of *Staphylococcus aureus*, *Escherichia coli*, *Salmonella*, and *Listeria monocytogenes* for the storage duration of up to 12 weeks at ambient and low temperatures. The total bacterial count (TBC) and yeast and mold count (YMC) of all mayonnaise samples rose significantly (*p* < 0.05) with storage duration at both storage conditions (25°and 7 °C) (Table 3 and Table 4). As expected, considerable differences between samples stored in cold and ambient conditions were observed. At 7 °C, the microbial increase was reported only from the 10th week onwards, while at 25 °C, an increase occurred from the 4th week onwards. However, neither the use nor the concentration of flaxseed oil significantly affected microbiological growth. According to Codex Stan. [44], the acceptable levels for TBC and YMC are 100 cfu/g and 50 cfu/g, respectively.

Pathogenic bacteria’s ability to survive in acidic environments poses a substantial food safety risk, which should be considered while producing new foods. *Lactobacilli*, an acid-tolerant microbe, can cause undesired alterations in food products and negatively impact sensory quality and shelf life [45]. *Lb. brevis*, *Lb. casei*, *Lb. plantarum*, *Lb. buchneri*, and *Lb. fructivorans* are the most common *lactobacillus* species that cause mayonnaise spoiling [46]. Acetic acid-resistant yeasts, such as *Z. bailii* and *Pichia membranaefaciens*, cause the most deterioration in mayonnaise and dressings. These bacteria may thrive in a 3% acetic acid medium as well as a weak acid medium. *Z. rouxii*, *Saccharomyces cerevisiae*, and *Candida magnolia* are the most common yeasts that cause mayonnaise deterioration. *Issatchenkia orientalis, C. inconspicua, C. parapsilosis, S. exiguous, S. dairensis, Debaryomyces hansenii, Rhodotorula mucilaginosa*, and *Torulopsis cutaneum* are among the yeasts that can deteriorate mayonnaise [47]. Molds rarely degrade mayonnaise, which contains acetic acid [48]. As determined in prior research, lactobacilli were commonly isolated from ruined mayonnaise and comparable products. The acidic state, which is present in mayonnaise and hinders the growth of most microorganisms, is a crucial element.

## 4. Conclusions

Flaxseed oil is one of the best plant-based sources of omega-3 fatty acids, mainly α-linolenic acid. Replacing soybean oil with 20–40% flaxseed oil in mayonnaise resulted in a healthier product, even at the lowest inclusion level, containing lower saturated fat and higher polyunsaturated fatty acids, particularly α-linolenic acid, without affecting the sensory properties. Thus, consuming the “new” mayonnaise will allow for higher ALA contribution and health benefits for consumers. Furthermore, this replacement positively affected the oxidative stability during 12 weeks of storage at 25 and 7 °C. These findings open prospects for producing foods, such as mayonnaise, with functional and healthy properties based on flaxseed oil, especially with the increase in omega-3 fatty acids and decrease in atherogenicity and thrombogenicity indices as observed in the current study.

## Figures and Tables

**Table 2:** Sensory results of mayonnaise samples.

	Quality Attributes of Mayonnaise Samples *					
	Appearance	Color	Taste	Consistency	Spread Ability	Overall Acceptability
Control sample	9.8 ^a^ ± 0.16	9.9 ^a^ ± 0.16	9.7 ^a^ ± 0.24	9.6 ^a^ ± 0.32	9.7 ^a^ ± 0.24	9.8 ^a^ ± 0.16
MFXS1	9.7 ^a^ ± 0.24	9.8 ^a^ ± 0.16	9.7 ^a^ ± 0.24	9.7 ^a^ ± 0.24	9.6 ^a^ ± 0.32	9.7 ^a^ ± 0.24
MFXS2	9.5 ^a^ ± 0.40	9.5 ^a^ ± 0.40	9.6 ^a^ ± 0.40	9.5 ^a^ ± 0.32	9.7 ^a^ ± 0.24	9.7 ^a^ ± 0.24
MFXS3	9.5 ^a^ ± 0.40	9.5 ^a^ ± 0.40	9.5 ^a^ ± 0.40	9.5 ^a^ ± 0.40	9.7 ^a^ ± 0.24	9.5 ^a^ ± 0.40

* Ten-point hedonic scale, with 1 indicating extreme dislike, 5 indicating neither like nor dislike, and 10 indicating extreme like. Values are the mean ± std. deviation. Values with different small letters in the same column are significantly different (*p* < 0.05). Control sample: 70% soy bean oil, MFXS1: 20% flaxseed oil and 50% soy bean oil, MFXS2: 30% flaxseed oil and 40% soy bean oil, and MFXS3: 40% flaxseed oil and 30% soy bean oil.

**Table 3:** The total bacterial counts (cfu/g) of mayonnaise samples during ambient and cold storage.

Storage Period (Week)		The Total Bacterial Count (cfu/g) of the Mayonnaise Samples *						
	Ambient Storage (25 ± 5 °C)				Cold Storage (7 ± 2 °C)			
	Control	MFXS1	MFXS2	MFXS3	Control	MFXS1	MFXS2	MFXS3
0	<10	<10	<10	<10	<10	<10	<10	<10
2	<10	<10	<10	<10	<10	<10	<10	<10
4	10 ^e^ ± 2.00	10 ^e^ ± 5.00	10 ^e^ ± 1.00	10 ^e^ ± 6.00	<10	<10	<10	<10
6	20 ^d^ ± 2.00	20 ^d^ ± 4.00	20 ^d^ ± 1.00	20 ^d^ ± 2.00	<10	<10	<10	<10
8	30 ^c^ ± 5.00	30 ^c^ ± 2.00	30 ^c^ ± 1.00	30 ^c^ ± 2.00	<10	<10	<10	<10
10	40 ^Ab^ ± 2.00	40 ^Ab^ ± 5.00	40 ^Ab^ ± 1.00	40 ^Ab^ ± 3.00	10 ^Bb^ ± 2.00	10 ^Bb^ ± 1.00	10 ^Bb^ ± 3.00	10 ^Bb^ ± 4.00
12	50 ^Aa^ ± 1.00	50 ^Aa^ ± 2.00	50 ^Aa^ ± 3.00	50 ^Aa^ ± 5.00	20 ^Ba^ ± 2.00	20 ^Ba^ ± 1.00	20 ^Ba^ ± 3.00	20 ^Ba^ ± 4.00

* Control sample: 70% soy bean oil, MFXS1: 20% flaxseed oil and 50% soy bean oil, MFXS2: 30% flaxseed oil and 40% soy bean oil, and MFXS3: 40% flaxseed oil and 30% soy bean oil. Values with different small letters in the same column or capital letters in the same row are significantly different (*p* < 0.05).

**Table 4:** Yeast and mold counts (cfu/g) of mayonnaise samples during ambient and cold storage.

Storage Period (Week)		Yeast and Mold Count (cfu/g) of the Mayonnaise Samples *						
	Ambient Storage (25 ± 5 °C)				Cold Storage (7 ± 2 °C)			
	Control	MFXS1	MFXS2	MFXS3	Control	MFXS1	MFXS2	MFXS3
0	<10	<10	<10	<10	<10	<10	<10	<10
2	<10	<10	<10	<10	<10	<10	<10	<10
4	10 ^c^ ± 2.00	10 ^c^ ± 1.00	10 ^c^ ± 3.00	10 ^c^ ± 4.00	<10	<10	<10	<10
6	10 ^c^ ± 2.00	10 ^c^ ± 1.00	10 ^c^ ± 3.00	10 ^c^ ± 4.00	<10	<10	<10	<10
8	20 ^b^ ± 2.00	20 ^b^ ± 5.00	20 ^b^ ± 1.00	20 ^b^ ± 2.00	<10	<10	<10	<10
10	30 ^a^ ± 2.00	30 ^a^ ± 1.00	30 ^a^ ± 2.00	30 ^a^ ± 3.00	<10	<10	<10	<10
12	30 ^Aa^ ± 3.00	30 ^Aa^ ± 1.00	30 ^Aa^ ± 5.00	30 ^Aa^ ± 2.00	10 ^Ba^ ± 2.00	10 ^Ba^ ± 5.00	10 ^Ba^ ± 1.00	10 ^Ba^ ± 3.00

* Control sample: 70% soy bean oil, MFXS1: 20% flaxseed oil and 50% soy bean oil, MFXS2: 30% flaxseed oil and 40% soy bean oil, and MFXS3: 40% flaxseed oil and 30% soy bean oil. Values with different small letters in the same column or capital letters in the same row are significantly different (*p* < 0.05).

## Data Availability

The data are available from the corresponding author.
